# Hemostatic Testing in Critically Ill Infants and Children

**DOI:** 10.3389/fped.2020.606643

**Published:** 2021-01-08

**Authors:** Alison B. Nair, Robert I. Parker

**Affiliations:** ^1^Pediatric Critical Care Medicine, University of California, San Francisco, San Francisco, CA, United States; ^2^Pediatric Hematology/Oncology, Renaissance School of Medicine, Stony Brook University, Stony Brook, NY, United States

**Keywords:** pediatric, developmental hemostasis, diagnosis, coagulopathy, viscoelastic

## Abstract

Children with critical illness frequently manifest imbalances in hemostasis with risk of consequent bleeding or pathologic thrombosis. Traditionally, plasma-based tests measuring clot formation by time to fibrin clot generation have been the “gold standard” in hemostasis testing. However, these tests are not sensitive to abnormalities in fibrinolysis or in conditions of enhanced clot formation that may lead to thrombosis. Additionally, they do not measure the critical roles played by platelets and endothelial cells. An added factor in the evaluation of these plasma-based tests is that in infants and young children plasma levels of many procoagulant and anticoagulant proteins are lower than in older children and adults resulting in prolonged clot generation times in spite of maintaining a normal hemostatic “balance.” Consequently, newer assays directly measuring thrombin generation in plasma and others assessing the stages hemostasis including clot initiation, propagation, and fibrinolysis in whole blood by viscoelastic methods are now available and may allow for a global measurement of the hemostatic system. In this manuscript, we will review the processes by which clots are formed and by which hemostasis is regulated, and the rationale and limitations for the more commonly utilized tests. We will also discuss selected newer tests available for the assessment of hemostasis, their “pros” and “cons,” and how they compare to the traditional tests of coagulation in the assessment and management of critically ill children.

## Introduction

Hemostatic dysfunction and resultant pathologic bleeding and/or thrombosis is a common complication of critical illness in children. There are several important considerations of this dysfunction that are imperative for accurate diagnosis and clinical management in the pediatric population. First, the hemostatic system changes and develops from birth to infancy, childhood, adolescence, and ultimately adulthood. Understanding this evolution is important in the interpretation of physiologic and pathologic hemostasis. Second, traditional tests of hemostasis have focused on platelet count and plasma-based measurements of clot formation. There are limitations in this traditional approach including lack of consideration for (1) platelet function and activity, (2) the imperfect sensitivity of plasma-based tests, particularly to abnormalities in fibrinolysis and conditions of enhanced clot formation, (3) the critical role of the endothelium in hemostasis and crosstalk between the endothelial, inflammatory, and coagulation systems, and (4) the stages of clot formation and lysis over time. Newer studies address some of these challenges by directly measuring thrombin generation in plasma and assessing the stages of hemostasis in whole blood. Successful diagnosis and management of hemostatic dysfunction in critically ill children requires both an age-dependent understanding of clot formation and regulation of hemostasis as well as of the strengths and weaknesses of the tests employed.

## Hemostasis and the Regulation of Clot Formation

Hemostasis is the normal physiologic process by which blood is maintained in fluid state while also allowing for blood clot formation at the site of injury maintaining the integrity of the closed circulatory system after vascular damage (1). It is a highly complex series of interwoven processes that result in a rapid, localized, and highly regulated response. Key elements of hemostasis include (1) formation of the platelet plug (referred to as primary hemostasis), (2) soluble phase coagulation with propagation of clotting through the clotting cascade, (3) termination of clot formation, and (4) clot dissolution (fibrinolysis). Under physiologic conditions, a clot is formed at the site of injury to stop bleeding locally with clot lysis and tissue remodeling to follow (1). Abnormalities in any part of these processes can result in dysfunctional hemostasis manifest as abnormal bleeding or thrombosis.

The hemostatic process starts with injury to the vascular endothelium (2). The undisturbed endothelium has multiple guards against undesired coagulation by functioning to counteract platelet activation and aggregation, and to maintain blood fluidity (2). When the integrity of the endothelium is compromised, exposure of subendothelial elements trigger thrombus formation (3). The endothelium also responds to injury through vasoconstriction which results from impairment of the vasodilatory action of damaged endothelial cells as well as direct access of smooth muscle cells to locally generated vasoconstrictive agents (3).

Platelet activation and adhesion upon exposure to vascular injury occurs in a highly coordinated method involving tethering, rolling, activation, and firm adhesion (4, 5). The initial interactions between platelets and the extracellular matrix are highly dictated by local rheological conditions (4). Platelet activation is triggered by several highly adhesive macromolecules including collagen and von Willebrand factor (vWf) along with the weaker agonists adenosine diphosphate (ADP) and epinephrine (4–6). This activation is also triggered by thrombin (4, 5). As part of the platelet activation process, platelets undergo shape change and develop elongated pseudopods that aid in the subsequent adhesion process (7). Platelet adhesion is primarily mediated by attachment of the platelet surface receptor GPIb/IX/V complex to vWf in plasma and the subendothelial matrix (7). Further, platelet activation results in exposure and conformational change of the GPIIb/IIIa receptor on the platelet surface. The GPIIb/IIIa receptor then binds vWf and fibrinogen ultimately resulting in platelet-platelet cohesion (8, 9).

After platelet binding to subendothelial structures and subsequent signaling to the platelet cytoplasm, platelet granules fuse with the open canalicular system of the platelet membrane and empty their contents into the local environment. The paracrine and autocrine nature of these bioactive contents causes increased activation of nearby platelets in both number and degree (4). This results in secondary secretion and significant amplification of the platelet activation and adhesion processes. Alpha (α)granules contain a heterogeneous complement of proteins that effect multiple biologic systems beyond primary hemostasis and coagulation including inflammation, angiogenesis, wound healing, and others (4). Important to formation of the platelet plug is the α-granule release of vWf which further increases formation of the platelet scaffold via GPIIb/IIIa and GPIb/IX/V as well as release of fibrinogen which crosslinks with GPIIb/IIIa and provides an additional source of fibrinogen to what is present in the plasma (4, 10). Dense (δ)-granules secrete ADP and serotonin which stimulate and recruit additional platelets (11). Finally, platelet procoagulant activity is an important facet of platelet activation. This procoagulant activity includes both exposure of procoagulant phospholipids such as phosphatidylserine and the ensuing formation of enzyme complexes on the platelet surface essential to the clotting cascade (12).

**Figure 1:**
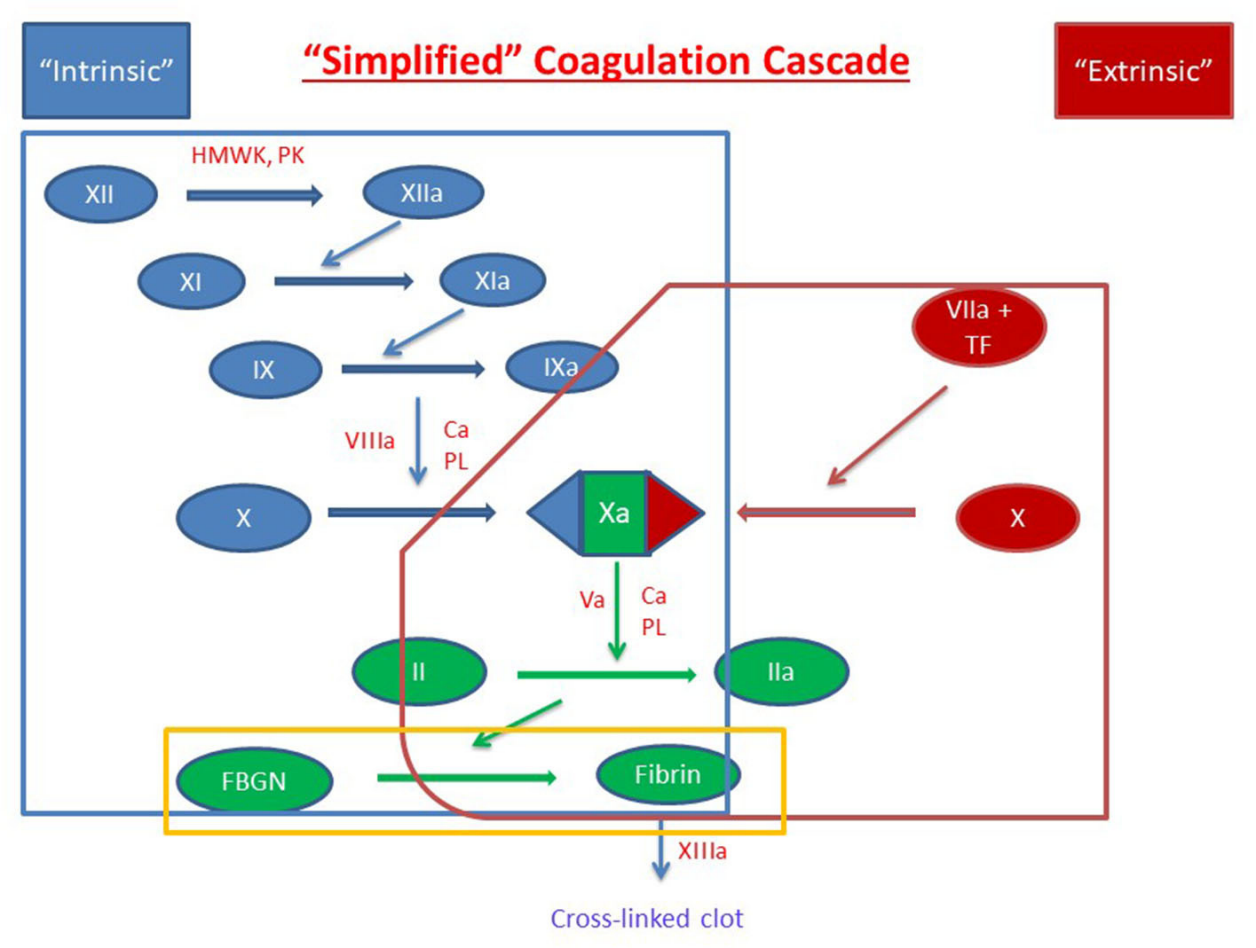
Simplified Coagulation Cascade. Clot formation as activated through the extrinsic pathway (red), the intrinsic pathway (blue) and the common pathway (green). Components of these pathways are measured by prothrombin time, activated partial thromboplastin time, and thrombin time are circled in red, blue, and yellow, respectively. Factors are indicated with a roman numeral and an “a” if activated. Abbreviations: Ca^2+^, calcium ions; FBGN, fibrinogen; HMWK, high-molecular-weight kininogen; PL, platelet membrane phospholipid; PK, prekallikrein; TF, tissue factor.

**Figure 2:**
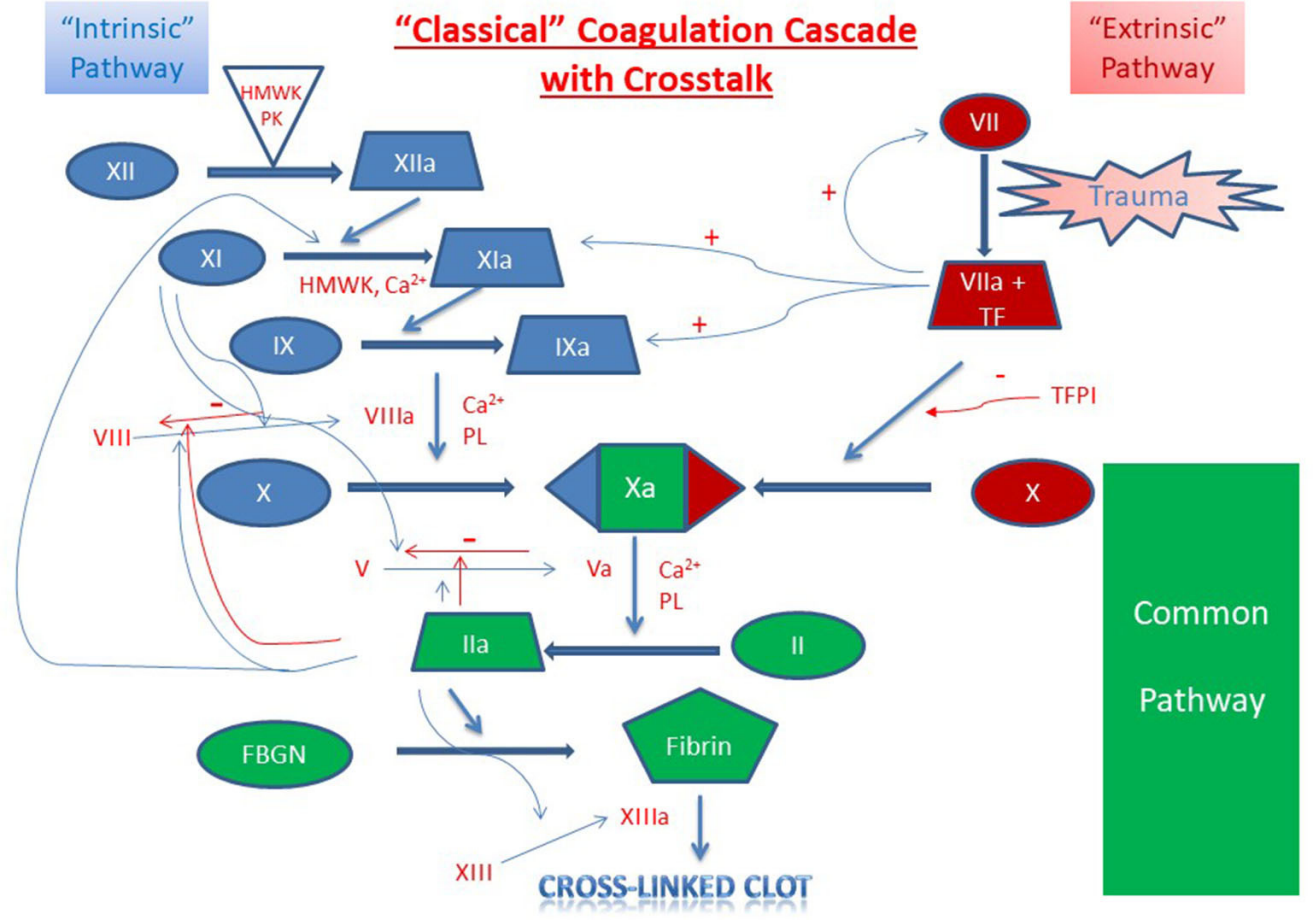
Classical Coagulation Cascade with Crosstalk. Clot formation as activated through the extrinsic pathway (red), intrinsic pathway (blue) and the common pathway (green). Crosstalk and feedback between pathways is shown in blue if positive and red if negative. Factors are indicated with a roman numeral and an “a” if activated. Abbreviations: Ca^2+^, calcium ions; FBGN, fibrinogen; HMWK, high-molecular-weight kininogen; PL, platelet membrane phospholipid; PK, prekallikrein; TF, tissue factor; TFPI, tissue factor pathway inhibitor.

The soluble phase of clotting primarily involves the clotting cascade (Figures 1, 2) (13). In general, the clotting cascade involves successive activation of a series of proenzymes or zymogens into active enzymes resulting in an incremental but amplified clotting response. The clotting cascade is classically described by the intrinsic, extrinsic, and common pathway, though further investigation has revealed these pathways to be much more complex and interrelated (Figure 1) (13).

The clotting cascade is initiated through the extrinsic pathway and starts with exposure of tissue factor (TF) at the site of injury. Blood is exposed to TF either directly by the subendothelial matrix or by cytokine-induced expression on endothelial cells or activated monocytes (14, 15). Platelets may also generate their own TF in proximity to the injured vessel (16). TF operates as a cofactor in the activation of factor VII and together these two factors form the extrinsic tenase multimeric complex which goes on to activate factors X and IX to factors Xa and IXa, respectively (17, 18).

The intrinsic or contact activation pathway is initiated through the interaction of negatively charged surfaces resulting in the activation of factor XII, high-molecular-weight kininogen, and plasma kallikrein among others (13). Activated factor XII in conjunction with high-molecular-weight kininogen activate factor XI which then activates factor IX (19). Activated factor IX forms a multimeric complex with activated factor VIII, which has been activated by factor X and thrombin produced in the extrinsic pathway. This multimeric complex, referred to as intrinsic tenase, subsequently activates sufficient factor X for clot formation (13, 19). This process is amplified because (1) factor VIII is activated by both activated factor X and thrombin and (2) activated factor IX is further activated by the thrombin-induced activation of factor XI (19, 20). As a result, there is a progressive increase in factor VIII and factor IX activation as factor Xa and thrombin are formed. Through these mechanisms, sustained and amplified generation of thrombin is achieved through the intrinsic pathway. Of note, while more activated factor X is generated through the intrinsic pathway due to amplifying steps in the cascade, the extrinsic pathway is physiologically more important clinically (21).

From there, both the extrinsic and intrinsic pathway proceed to the common pathway. Factor V, which is released from platelet α-granules, is cleaved by thrombin to form activated factor V (22, 23). Activated factors X and V bind on the platelet phospholipid surface to form the prothrombinase complex which converts prothrombin (factor II) to thrombin (activated factor II, factor IIa) (24). Thrombin then converts fibrinogen to fibrin and activated factor XIII crosslinks overlapping fibrin stands which leads to stabilization of the clot (25).

Intrinsic to the built-in amplification systems of formation of the platelet plug and the coagulation cascade, hemostasis also requires highly regulated mechanisms of control of clot extension and termination of clot formation (Figure 2). Systemic control of this localized response is modulated through several mechanisms including (1) dilution of procoagulants in the bloodstream, (2) elimination of activated factors through the reticuloendothelial system, and (3) control through antithrombotic pathways anchored on vascular endothelial cells (1). Physiologic inhibitors of coagulation include tissue factor pathway inhibitor (TFPI) which inhibits TF-mediated and factor VIIa-mediated factor X activation, and C1 esterase inhibitor which inhibits activated factor XII, plasma kallikrein, and several complement proteases (26, 27).

Cessation of clot formation is critical in maintaining a targeted response and mediating the extent of the clot. Clot termination involves two key components, antithrombin (antithrombin-III, AT) and the protein C pathway. AT is a serine protease inhibitor that neutralizes many enzymes in the clotting cascade by irreversibly binding to them (28). Its function is enhanced by endogenous heparins and heparan sulfate, which induce a conformational change and increase its affinity for thrombin approximately 300-fold (28–30). Vascular endothelial cells are coated with activated AT and therefore equipped to rapidly inactivate any excess generated thrombin (29, 30). The protein C pathway is initiated through the binding of thrombin to the endothelial membrane-expressed thrombomodulin which induces the ability of the thrombomodulin-thrombin complex to activate protein C (31, 32). In association with protein S, activated protein C then proteolytically inactivates activated factors V and VIII (33, 34).

Also important to the limitation of clot formation is the regulation of vascular and platelet reactivity which is primarily modulated through prostacyclin, thromboxane, and nitric oxide. Specifically, undisturbed endothelial cells adjacent to endothelial injury release arachidonic acid which is subsequently converted to thromboxane A2 by cyclooxygenase-1 in platelets and prostacyclin via cyclooxygenase-1 on the endothelium (35). Prostacyclin blocks platelet aggregation and counteracts thromboxane A2-mediated vasoconstriction (36). In addition to its vasodilatory effects, nitric oxide inhibits platelet adhesion and aggregation (37). In fact, platelets may enhance their synthesis of nitric oxide in the setting of platelet adhesion to collagen providing negative feedback to limit excessive platelet adhesion and vasoconstriction at the site of injury (38).

Clot removal following hemostasis is achieved through fibrinolysis. Central to this process, plasmin is formed from the cleavage of plasminogen by bound fibrin and tissue-type plasminogen activator (38, 39). Urokinase is a secondary plasminogen activator, primarily acting in the extravascular compartment (39). Once formed, plasmin cleaves fibrinogen, fibrin, activated factor XIII, as well as a variety of plasma proteins and other clotting factors (39–41). Fibrinolytic activity can be generated either on the surface of the fibrin-containing thrombus or on cells that express profibrinolytic receptors (38). Plasmin activity is regulated by vascular endothelial cells that secrete both serine protease plasminogen activators and plasminogen activator inhibitors (42). Fibrinolysis works in conjunction with wound healing and tissue remodeling to restore vessel patency following injury.

**Figure 3:**
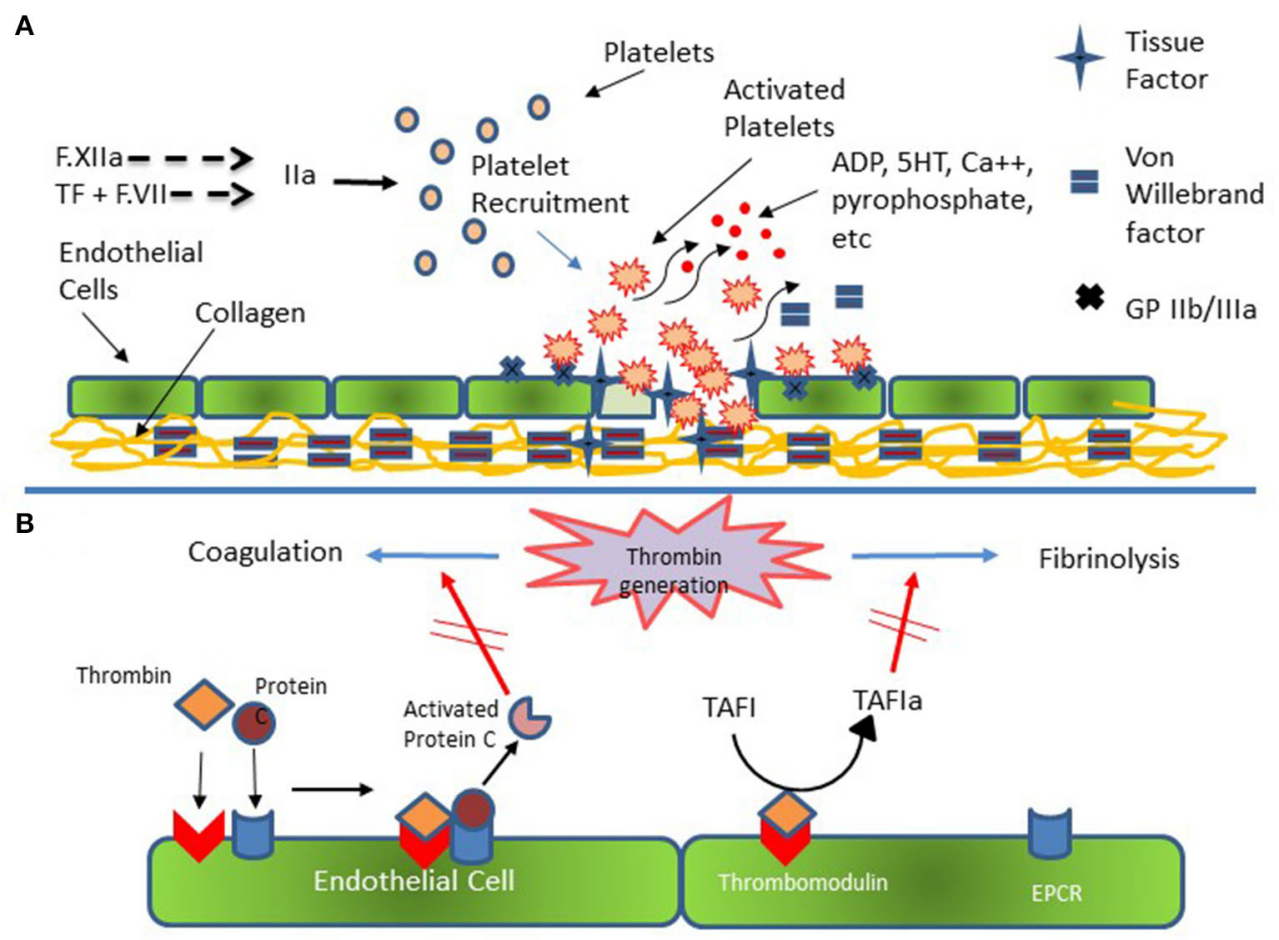
Platelet and Endothelial Cell Interactions in Hemostasis. **(A)** Injured endothelial cells elaborate tissue factor (TF) with consequent activation of the coagulation cascade and generation of thrombin (F.IIa). Thrombin activates platelets which subsequently adhere to injured endothelial cells and to subendothelial collagen via specific receptors on endothelial and platelet membrane and via von Willebrand [vWf]. Additional platelets are recruited to the area of injury with subsequent formation of platelet plug. **(B)** Thrombin generation results in activation of coagulation and fibrinolysis. Thrombin bound to endothelial cell surface thrombomodulin catalyzes the conversion of Protein C bound to endothelial cell protein C receptor (EPCR). Activated Protein C then down regulates further thrombin formation by inactivating activated F.VIII and activated F.V. Additionally, Thrombomodulin bound thrombin catalyzes Thrombin Activatable Fibrinolysis Inhibitor (TAFI) to its active form (TAFIa) which damps the activation of fibrinolysis thereby promoting clot formation and stability.

**Figure 4:**
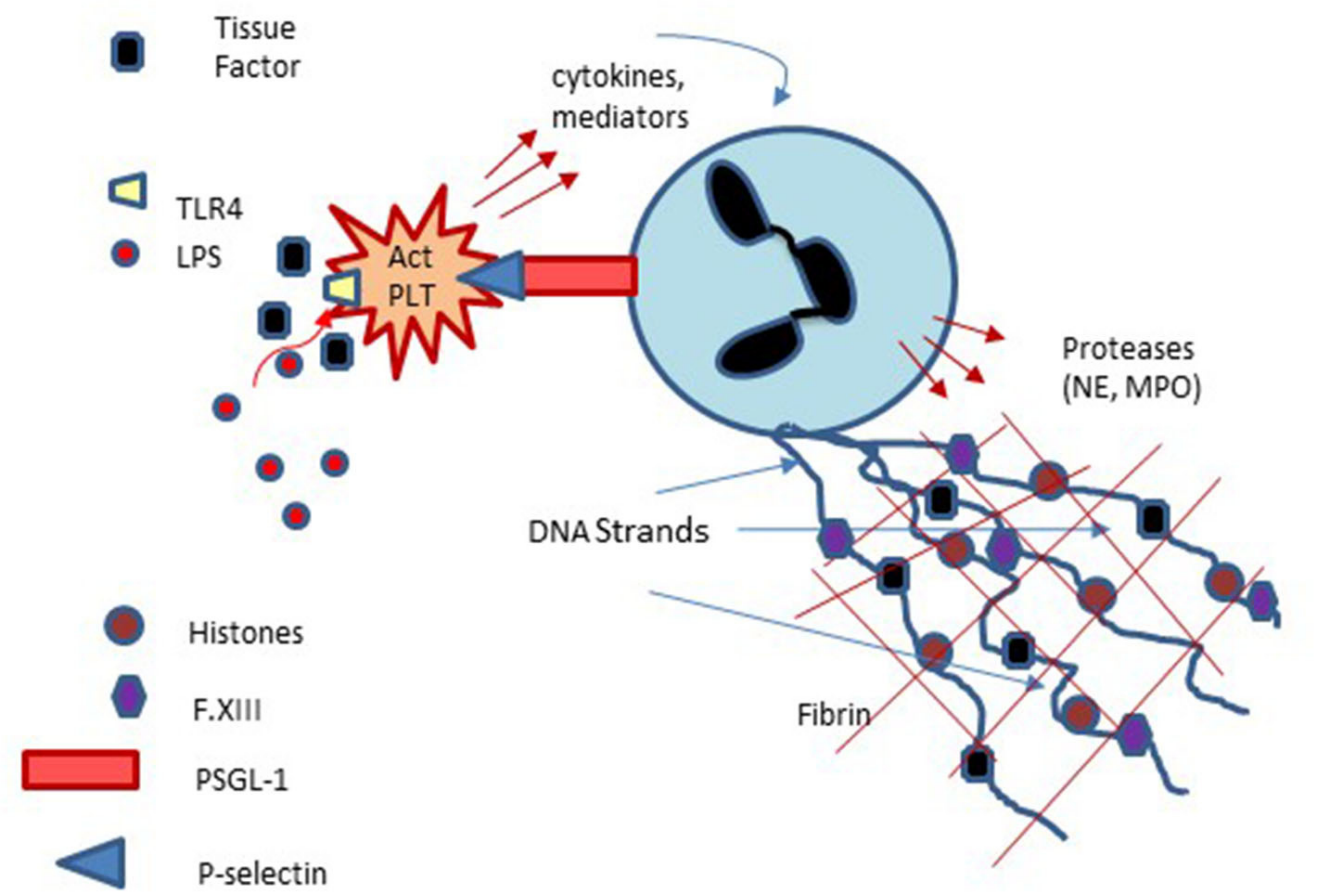
Platelet involvement in NETosis and Immunothrombosis: Platelets interact with neutrophils via platelet membrane expressed P-selectin and P-selectin glycoprotein ligand-1 (PSGL-1) expressed on neutrophils with subsequent enhancement of neutrophils, extrusion of neutrophil DNA and formation of Neutrophil Extracellular Traps (NETs) which can serve as a nidus of thrombus formation. Platelet activation via lipopolysaccharide (LPS) binding to platelet Toll-like receptors may also participate in NET formation and NETosis.

The above discussion focuses primarily on plasma proteins with only limited mention of the role cellular components play in hemostasis. Indeed, a major limitation of plasma-based coagulation tests is that they do not take into consideration the myriad roles platelets, endothelial and leukocytes play in this process (43). Platelets are recruited to areas of vascular injury and adhere to endothelial cells and subendothelial structures (e.g., collagen) via specific receptors on these structures, a process augmented through binding to von Willebrand factor (vWf), thereby forming a platelet plug (Figure 3A). As noted, endothelial cells are critical in modulating the balance between activation of coagulation and fibrinolysis mediated through the binding of thrombin to thrombomodulin (Figure 3B). Additionally, platelet-neutrophil interactions are important in the formation of Neutrophil Extracellular Traps (NETs) and the process of NETosis which can induce a process referred to as immunothrombosis (Figure 4) (44, 45).

## Developmental Hemostasis

Developmental hemostasis describes the physiologic changes that occur with an increase in age. These changes are particularly relevant in the pediatric population from birth through infancy when plasma levels of several important procoagulant and anticoagulant factors rapidly increase or decrease to normal adult levels. Because of the dynamic development of the hemostatic system, an understanding of age-specific physiology and quantification of normal reference ranges for coagulation parameters is of particular importance in pediatric medicine.

Early study of developmental hemostasis included the establishment of reference ranges for basic global measures of coagulation in infants using cord blood (46, 47). Andrew et al. were the first to define the field of developmental hemostasis in a series of articles describing reference values for coagulation parameters in (1) healthy term infants from birth to 6 months of age, (2) healthy preterm infants from birth to 6 months of age, and (3) healthy children and adolescents between 1 and 16 years of age (Table 1) (48–50). This work defined normal values of global measures of coagulation, individual factors that facilitate clot formation, and inhibitors of coagulation (Table 2).

**Table 1:** Study details of the Andrew et al. series describing the developing hemostatic system during infancy, childhood, and adolescence (45–47).

**Year**	**Study details**	**Coagulation parameters**	**Notable findings**
1987 (45)	• Study population: Consecutively born term infants (37–42 weeks) at a single center over a 3-month period • Sample size: 118 total infants, 61–77 at each study timepoint • Timepoints: Day 1, 5, 30, 90, and/or 180 after birth • Relevant methods: ACL analyzer, chromogenic assays, ELISA	All studies: • Global: aPTT, fibrinogen, PT • Coagulation: FII, FV, FVII, FVIII, FIX, FX, FXI, FXII, FXIIIa, FXIIIb, HMWK, PK, vWf • Fibrinolysis: α_2_-AP, plasminogen • Inhibitors: α_1_-AT, α_2_-M, AT, C1-INH, HCII, protein C, protein S	General findings: • Normal values from birth to 6 months old in the healthy term infant and preterm infant, and from 1 to 16 years old in the child and adolescent were determined • Coagulation tests varied with age and different parameters showed different patterns of maturation Selected specific findings: • Global: • aPTT was prolonged at birth, remained prolonged throughout the postnatal period in preterm infants, and reached adult levels by 3 months in term infants • PT demonstrated variability at birth and shortened to adult levels by 1 month in both term and preterm infants • Despite an aPTT and PT similar to adults, BT was prolonged in children and adolescents at all ages compared to adults
1988 (46)	• Study population: Preterm infants (born at 30–36 weeks) at a single center over a 3.25-year period • Sample size: 137 total infants, 40 to 96 at each study timepoint • Timepoints: Day 1, 5, 30, 90, and/or 180 after birth • Relevant methods: ACL analyzer, chromogenic assays, ELISA	Term/preterm infant studies: • Global: TTChildren/adolescents study: • Global: BT, INR • Fibrinolysis: PAI, TPA	• Coagulation: • Vitamin K-dependent factors (FII, FVII, FIX, FX, protein C, and protein S) were low throughout the postnatal period in term and preterm infants as well as in children and adolescents through age 16 compared to adults • FXI, FXII, HWMK, and PK were lower in term and preterm infants in the postnatal period compared to adults • FXI and FXII decreased at 11 to 16 years old compared to adults • FV decreased throughout childhood and was lowest in adolescence • FVIII and vWf were similar in term and preterm infants and reached near or above adult values by 6 months • Factor XIIIb was elevated in early childhood and decreased with age
1992 (47)	• Study population: Healthy children and adolescents (1–16 years old) at a single center over a 2-year period • Sample size: 246 total patients, 4–7 at each age • Relevant methods: ACL analyzer, chromogenic assays, and ELISA		• Fibrinolysis: • Plasminogen gradually rose to near adult levels by 6 months in term infants but remained persistently low in preterm infants • In children and adolescents, TPA levels were low and PAI levels were increased compared to adults • Inhibitors: • All inhibitors were low at birth in term and preterm infants but rose to or near to adult levels by 6 months of age • In preterm infants, α_2_-M which was above adult levels at birth and rose to twice adult levels by 6 months of age • Protein C and S remained below adult levels until adolescence

*aPTT, activated partial thromboplastin time; α_1_-AT, α_1_-antitrypsin; α_2_-M, α_2_-macroglobulin; α_2_-AP, α_2_-antiplasmin; AT, antithrombin or antithrombin III; BT, bleeding time; C1-INH, C1 inhibitor or C1 esterase inhibitor; ELISA, enzyme-linked immunosorbent assay; F, factor; INR, international normalized ratio; HCII, heparin cofactor II; HMWK, high-molecular-weight kininogen; PK, prekallikrein; PAI, plasminogen activator inhibitor; PT, prothrombin time; TPA, tissue plasminogen activator; TT, thrombin clotting time; vWf, von Willebrand factor*.

**Table 2:** Mean values and 95% confidence intervals of key hemostasis measures in term infants, preterm infants, children, and adolescents compared to normal adult values based on the Andrew et al. series (46–48).

**Test**	**GA**	**1 day**	**30 days**	**180 days**	**1–5 years**	**6–10 years**	**11–16 years**	**Adult**
PT (s)	Term	13.0 (12.6–13.4)	11.8 (11.5–12.1)	12.3 (12.1–12.5)				12.4 (10.8–13.9)^+^
	Preterm	13 (10.6–16.2)	11.8 (10–13.6)	12.5 (10–15)				
					11 (10.6–11.4)	11.1 (10.1–12.1)	11.2 (10.2–12)	12 (11–14)^++^
aPTT (s)	Term	42.9 (41.4–44.4)^*^^#^	40.4 (38.6–42.2)^*^	35.5 (34.4–36.6)				33.5 (26.6–40.3)^+^
	Preterm	53.6 (27.5–79.4)^*^^#^	44.7 (26.9–62.5)^*^	37.5 (21.7–53.3)				
					30 (24–36)	31 (26–36)	32 (26–37)	33 (27–40)^++^
TT (s)	Term	23.5 (22.9–24.1)	24.3 (23.6–25)	25.5 (24.6–26.4)				25 (19.7–30.3)
	Preterm	24.8 (19.2–30.4)	24.4 (18.8–29.9)	25.2 (18.9–31.5)		
Fibrinogen (g/L)	Term	2.83 (2.68–2.98)^#^	2.7 (2.57–2.83)	2.51 (2.32–2.7)	2.76 (1.7–4.05)	2.79 (1.57–4)	3 (1.54–4.48)	2.78 (1.56–4)
	Preterm	2.43 (1.5–3.73)^#^	2.54 (1.5–4.14)	2.28 (1.5–3.6)^*^				
BT					6 (2.5–10)^*^	7 (2.5–13)^*^	5 (3–8)^*^	4 (1–7)
Factor II (u/mL)	Term	0.48 (0.45–0.51)^*^	0.68 (0.64–0.72)^*^^#^	0.88 (0.84–0.92)^*^	0.94 (0.71–1.16)^*^	0.88 (0.67–1.07)^*^	0.83 (0.61–1.04)^*^	1.08 (0.7–1.46)
	Preterm	0.45 (0.2–0.77)^*^	0.57 (0.36–0.95)^*^^#^	0.87 (0.51–1.23)^*^				
Factor V (u/mL)	Term	0.72 (0.68–0.77)^*^^#^	0.98 (0.94–1.02)^*^	0.91 (0.86–0.96)^*^	1.03 (0.79–1.27)	0.9 (0.63–1.16)^*^	0.77 (0.55–0.99)^*^	1.06 (0.62–1.5)
	Preterm	0.88 (0.41–1.44)^#^	1.02 (0.48–1.56)^*^	1.02 (0.58–1.46)^*^				
Factor VII (u/mL)	Term	0.66 (0.61–0.71)^*^	0.90 (0.84–0.96)^*^	0.87 (0.81–0.93)^*^	0.82 (0.55–1.16)^*^	0.85 (0.52–1.2)^*^	0.83 (0.58–1.15)^*^	1.05 (0.67–1.43)
	Preterm	0.67 (0.21–1.13)^*^	0.83 (0.21–1.45)^*^	0.99 (0.47–1.51)				
Factor VIII (u/mL)	Term	1 (0.9–1.1)	0.91 (0.83–0.99)^#^	0.73 (0.68–0.78)^*^^#^	0.9 (0.59–1.42)	0.95 (0.58–1.32)	0.92 (0.53–1.31)	0.99 (0.5–1.49)
	Preterm	1.11 (0.50–2.13)	1.11 (0.5–1.99)^#^	0.99 (0.5–1.87)^#^				
vWf (u/mL)	Term	1.53 (1.32–1.74)^*^	1.28 (1.1–1.46)^*^	1.07 (0.94–1.2)^*^	0.82 (0.6–1.2)	0.95 (0.44–1.44)	1 (0.46–1.53)	0.92 (0.5–1.58)
	Preterm	1.36 (0.78–2.1)^*^	1.36 (0.66–2.16)^*^	0.98 (0.54–1.58)				
Factor XI (u/mL)	Term	0.38 (0.35–0.42)^*^^#^	0.53 (0.5–0.56)^*^^#^	0.86 (0.79–0.93)^*^	0.97 (0.56–1.5)	0.86 (0.52–1.2)	0.74 (0.5–0.97)^*^	0.97 (0.67–1.27)
	Preterm	0.3 (0.08–0.52)^*^^#^	0.43 (0.15–0.71)^*^^#^	0.78 (0.46–1.1)^*^				
Factor XII (u/mL)	Term	0.53 (0.48–0.58)^*^^#^	0.49 (0.45–0.53)^*^	0.77 (0.72–0.82)^*^	0.93 (0.64–1.29)	0.92 (0.6–1.4)	0.81 (0.34–1.37)^*^	1.08 (0.52–1.64)
	Preterm	0.38 (0.1–0.66)^*^^#^	0.43 (0.11–0.75)^*^	0.82 (0.22–1.42)^*^				
Plasminogen CTA, (u/mL)	Term	1.95 (1.85–2.05)^*^^#^	1.98 (1.88–2.08)^*^	3.01 (2.9–3.12)^*^^#^				3.36 (2.48–4.24)^+^
	Preterm	1.7 (1.12–2.48)^*^^#^	1.81 (1.09–2.53)^*^	2.75 (1.91–3.59)^*^^#^				
					0.98 (0.78–1.18)	0.92 (0.75–1.08)	0.86 (0.68–1.03)^*^	0.99 (0.77–1.22)^++^
AT (u/mL)	Term	0.63 (0.6–0.66)^*^^#^	0.78 (0.74–0.82)^*^^#^	1.04 (1.01–1.07)^#^				1.05 (0.79–1.31)^+^
	Preterm	0.38 (0.14–0.62)^*^^#^	0.59 (0.37–0.81)^*^^#^	0.9 (0.52–1.28)^*^^#^				
					1.11 (0.82–1.39)	1.11 (0.9–1.31)	1.05 (0.77–1.32)	1 (0.74–1.26)^++^
Protein C (u/mL)	Term	0.35 (0.32–0.38)^*^^#^	0.43 (0.4–0.46)^*^^#^	0.59 (0.56–0.62)^*^	0.66 (0.4–0.92)^*^	0.69 (0.45–0.93)^*^	0.83 (0.55–1.11)^*^	0.96 (0.64–1.28)
	Preterm	0.28 (0.12–0.44)^*^^#^	0.37 (0.15–0.59)^*^^#^	0.57 (0.31–0.83)^*^				
Protein S (u/mL)	Term	0.36 (0.32–0.4)^*^^#^	0.63 (0.58–0.68)^*^	0.87 (0.83–0.92)				0.92 (0.6–1.24)^+^
	Preterm	0.26 (0.14–0.38)^*^^#^	0.56 (0.22–0.9)^*^	0.82 (0.44–1.2)^*^				
					0.86 (0.54–1.18)	0.78 (0.41–1.14)	0.72 (0.52–0.92)	0.81 (0.6–1.13)^++^

*aPTT, activated partial thromboplastin time; AT, antithrombin or antithrombin III; BT, bleeding time; PT, prothrombin time; TT, thrombin clotting time; vWf, von Willebrand factor*.

* *Values that differ statistically from adult values*.

# *Values that differ statistically in term and preterm infants*.

+ *Adult reference value specific for term/preterm infant studies*.

++ *Adult reference value specific for children/adolescents study*.

Overall, Andrew et al. found that coagulation tests varied with age and different parameters showed different patterns of maturation (Tables 1, 2) (48–50). At birth, plasma levels of many important coagulation factors were found to be around half of that found in adults. Full maturation to adult levels of these factors varied between a few months to 16 years of age (48–50). While there were continued increases in some factors through later childhood, most of these factors reached near normal adult levels by 6 months of age. Specifically, in both term and preterm infants the mean values for the vitamin K-dependent factors, contact factors, and selected inhibitors including antithrombin (AT), protein C and protein S were well-below adult values. In contrast, fibrinogen, factors V, VIII, and XIII, vWf and the inhibitors α_1_-antitrypsin, α_2_-antiplasmin, α_2_-macroglobulin, and C_1_ esterase inhibitor were noted to be at normal or somewhat supra-normal adult levels at birth. Globally, both prothrombin time (PT) and activated partial thromboplastin time (aPTT) were prolonged in neonates. While there was wide variation in normal PT in infancy, mean PT normalized to adult levels by 1 month of age whereas aPTT normalized by 6 months of age (48, 49). Differences between preterm and term infants tend to be present but relatively small (48, 49). By 6 months of age both preterm and full term infants exhibit equivalent levels of all but four components of the coagulation system, namely factor VIII (mean 0.99 in preterm compared to 0.73 in term), plasminogen (mean 2.75 in preterm compared to 3.01 in term), antithrombin (mean 0.9 in preterm compared to 1.04 in term), and heparin cofactor II (mean 0.89 in preterm compared to 1.2 in term) (49).

Subsequent studies went on to confirm the highly selective pattern of maturation of the coagulation system described by Andrew et al. using different analyzer and reagent combinations (Table 3) (51–55). Compared to Andrew et al. these studies report the same trends by age and value relative to adult values but establish different reference intervals due to use of different equipment and reagents instead of the manual methods employed by Andrew (51–55). In addition to updated analysis techniques, several studies have expanded the panel hemostatic markers (Table 3) (51–53). Flanders et al. described differences by sex in protein S levels with females having higher than adult values at all ages and males having higher than adult values only in the 16–17 year old age group (101–103% in females 7–17 years old vs. 98% in female adults; 116% in males 16–17 years old vs. 108% in male adults) (51).

**Table 3:** Key articles in developmental hemostasis following the Andrew et al. series (48–52).

**References**	**Study details**	**Coagulation parameters**	**Notable findings**
Flanders et al. (48)	• Study population: Healthy older children and adolescents (7–16 years old) at a single center • Sample size: 887 total patients, 75–245 patients in each 2–3 year age cohort • Relevant methods: STA-R coagulation analyzer	• Global: Fibrinogen • Coagulation: FII, FV, FVII, FX, • Fibrinolysis: α_2_-AP, plasminogen • Inhibitors: AT, protein C, protein S	General findings: • Similar maturation patterns but different absolute values were identified compared to the Andrew et al. series Unique findings (median values): • Protein S: • Females: 101–103% between 7 and 17 years old compared to 98% in adults^*^ • Male: 116% at 16–17 years old compared to 108% in adults^*^
Monagle et al. (49)	• Study population: Newborns (day of life 1, 3), infants, children, and adolescents (1 month to 16 years old) at a single center • Sample size: 159 newborns and 458 patients 1 month to 16 years of age • Relevant methods: STA compact analyzer, chromogenic assays, functional clotting assays	• Global: aPTT, fibrinogen, INR, PT, TT • Coagulation: FII, FV, FVII, FVIII, FIX, FX, FXI, FXII • Fibrinolysis: D-dimer • Inhibitors: AT, protein C, protein S • Others: ETP, TFPI (free, total)	General findings: • While mirroring the trends described by Andrew et al. differences in the absolute values of coagulation assays with most measures proportionally increased in all age-groups were demonstrated Unique findings (mean values): • D-dimers: • 1.34–1.47 in neonates compared to 0.18 in adults^*^ • 0.25–0.27 between 1 and 16 years old compared to 0.18 in adults^*^ • ETP: • 4,429–5,363 between 1 month and 10 years old compared to 8,475 in adults^*^ • Free TFPI: • 7.13 between 1 month and 1 year old compared to 10.7 in adults^*^ • 6.69–7.66 between 6 and 16 years old compared to 10.7 in adults^*^
Appel et al. (50)	• Study population: Full term infants and children age 0–17 years old and adults between the ages of 20 and 49 years old • Sample size: 218 children and 52 adults • Relevant methods: Sysmex CA-1500 System, Behring BCS System	• Global: aPTT, BT, fibrinogen, PT, TT • Coagulation: FII, FV, FVII, FVIII, FIX, FX, FXI, FXII, FXIII, vWf • Fibrinolysis: α_2_-AP, D-dimer, plasminogen • Inhibitors: AT, protein C, protein S	General findings: • Differences in coagulation markers were most significant in infants <12 months compared to older children / adults • vWf antigen and activity were higher in infants compared to older children • Levels in non-O blood groups reached their nadir at 12 months then gradually increased toward adult levels, while these nadirs were less distinct in the O blood group Unique findings (median values using the BCS system): • vWf antigen: • 97% in non-O blood groups compared to 71% in the O blood group at 1–5 years old^#^ • 118% in non-O blood groups compared to 99% in O the blood group at >19 years old^#^ • vWF, ristocetin cofactor: • 82% in non-O blood groups compared to 66% in the O blood group at 1–5 years old^#^ • 106% in non-O blood groups compared to 82% in the O blood group at >19 years old^#^ • Factor VIII: • 121% in non-O blood groups compared to 100% in the O blood group at 1–5 years old^#^ • 105% in non-O blood groups compared to 90% in the O blood groups at 6–10 years old^#^ • 131% in non-O blood groups compared to 116% in the O blood group at >19 year old^#^
Attard et al. (51)	• Study population: Newborns (day of life 1, 3), infants, children, and adolescents (1 month to 16 years old) • Sample size: 120 patients • Relevant methods: ELISA	• Coagulation: FII, FV, FVII, FVIII, FIX, FX, FXI, FXII, FXIII • Fibrinolysis: Plasminogen • Inhibitors: AT, protein C, protein S (total, free)	General findings: • Combined results from the study cohorts demonstrated differences in line with prior studies along with a high level of interindividual variability in specific protein levels
Toulon et al. (52)	• Study population: Infants, children, and adolescents between 15 days and 17 years old across 7 study centers • Sample size: 1,437 total infants, children, and adolescents • Relevant methods: Clotting assays, latex agglutination, chromogenic analysis, colorimetric analysis	• Global: aPTT, fibrinogen, PT • Coagulation: FII, FV, FVII, FVIII, FIX, FX, FXI, FXII, FXIII, vWf • Fibrinolysis: D-dimer, plasminogen • Inhibitors: AT, protein C, protein S	General findings: • Values followed similar patterns as prior studies and were not significantly different between the seven study centers

*aPTT, activated partial thromboplastin time; α_2_-AP, α_2_-antiplasmin; AT, antithrombin or antithrombin III; BT, bleeding time; ETP, endogenous thrombin potential; F, factor; INR, international normalized ratio; PT, prothrombin time; TFPI, tissue factor plasmin inhibitor; TT, thrombin clotting time; vWf, von Willebrand factor*.

* *Indicates findings statistically significant from adult values*.

# *Indicates statistically significant findings between blood groups*.

Monagle et al. described elevated D-dimer, reduced TFPI, and reduced endogenous thrombin potential in healthy neonates and children compared to established adult values (52). Appel et al. described variation in development of vWf and factor VIII across blood groups, specifically (1) a higher median vWf antigen in non-O compared to O groups at 1–5 years old (97 vs. 71%) and >19 years old (118 vs. 99%), (2) a higher median ristocetin cofactor activity in non-O compared to O groups at 1–5 years old (82 vs. 66%) and >19 years old (106 vs. 82%), and (3) a higher median factor VIII in non-O compared to O groups at 1–5 years old (121 vs. 100%) and >19 years old (131 vs. 116%) (53). Attard et al. combined the results of several cohorts of infants at different ages and found maturation patterns that mirrored prior studies along with a high level of interindividual variability in protein levels (54). Finally, Toulon et al. looked across seven centers and found values that were not significantly different between centers when using the same analysis techniques and reagents (55). In contrast to other reports in which primarily activity of clotting proteins is measured, Attard tracks changes in antigenic levels of hemostatic factors over age (54). It is assumed that the relationship between antigen and activity is (relatively) fixed and that a decrease in antigen implies a commensurate decrease in activity with clinical decisions being made accordingly. However, one cannot rule out the possibility that changes in antigenic level or protein activity noted with age reflect an element of post-translational protein modification.

Taken together, these studies show that most coagulation parameters are highly dependent on age and undergo the greatest changes during the 1st year of life. These changes require the use of age-specific reference intervals in the clinical assessment of hemostasis and the diagnosis of pediatric bleeding and thrombotic disorders. Despite these age-dependent differences, the changing coagulation system throughout infancy, childhood, and adolescence should be viewed as physiologic, i.e., “balanced,” with decreased factor levels and prolonged PT and aPTT in infants compared to normal adolescents and adults not necessarily reflecting an increased risk for hemorrhage. In fact, the pediatric hemostatic system may favor a reduced risk of thrombosis without an increased risk of bleeding (52, 56). While generation of thrombin in infants has been shown to be less than that in adults, this is counterbalanced by the reduction in AT and the fact that that the major physiologic inhibitor of thrombin in infants, α_2_-macroglobulin, is less efficient than is AT. However, other studies have shown that thrombin generation (endogenous thrombin potential; ETP) is “normal” in infants if measured in the presence of added thrombomodulin and that thrombin generation in very pre-term newborns is not different than that in near term infants (57, 58). That infants exhibit a “balanced” hemostatic system is supported by clinical findings such as decreased thromboembolic disease following surgery or prolonged immobilization in children compared to adults with similar risk factors (52, 59, 60). That said, the molecular basis underlying age-related numerical changes in coagulation measures as well as the clinical manifestation of these changes is not fully understood. Animal studies have demonstrated structural differences in coagulation proteins in newborn compared to adult models but this work has yet to be translated to humans (61). Further translational study is necessary to elucidate the important connections between coagulation parameters and clinical thrombotic and bleeding risk.

## Key Laboratory Tests of Hemostasis

Evaluation of abnormalities in pediatric hemostasis requires (1) knowledge of different hemostatic testing options, (2) an understanding of the strengths and weaknesses of each test, and (3) an awareness of utility in the pediatric population, particularly in the context of the developmental hemostasis consideration discussed above. The following are key tests of pediatric hemostasis with specific focus on platelet evaluation, clotting assays, and global measures. Logistics of testing as well as strengths and weaknesses are summarized in Table 4.

**Table 4:** Overview of selected testing in hemostasis.

**Test category**	**Test**	**Blood volume required**	**Venue performed**	**Advantages and limitations**
Platelet evaluation	Platelet count (58–62)	• Clinical laboratory: 1 mL whole blood • Sample test volume: ≤ 250 μL whole blood	Clinical laboratory	Advantages: • Has common clinical use with validated standardized procedures • Is simple to perform either manually or through automated techniques Disadvantages: • Does not provide information about platelet function • Can be time intensive (primarily manual counts) • Overestimates when cellular debris or other cell populations are present • Underestimates in samples with enlarged platelets or platelet clumping • Has limited accuracy, particularly very low platelet counts (<10,000/μL)
	Light transmission aggregometry (57–70)	• Clinical laboratory: 20 mL whole blood • Sample test volume: 3–5 mL platelet-rich plasma or whole blood	Clinical laboratory	Advantages: • Tests specific platelet function responses to a panel of agonist • May be augmented with light scatter techniques to better capture the early phase of aggregation and assess aggregates of different sizes Disadvantages: • Is time and resource intensive • Requires special expertise and training to interpret • Requires large blood volumes, particularly for the pediatric population • May be unreliable if the initial specimen contains platelet aggregates
	Bleeding time (71–74)	NA	Point-of-care	Advantages: Evaluates *in vitro* platelet function through *in vivo* test Does not require specialized equipment to perform Disadvantages: Is time and resource intensive Requires an invasive procedure Requires specialized training to perform (manual method) Has variable reproducibility Has unclear ability to predict bleeding risk unless grossly abnormal
	PFA-100/PFA-200 (74–76)	• Clinical laboratory: 3–5 mL whole blood • Sample test volume: 1–3 mL whole blood	Point-of-care	Advantages: • May provide a more standardized approach than other platelet function testing • Measures platelets at high (physiologic) shear rates Disadvantages: • Is time and resource intensive • Provides unclear association of platelet function and bleeding risk
	Cone and plate analyzer (74, 77)	• Clinical laboratory: 3–5 mL whole blood • Sample test volume: 150–250 μL whole blood	Clinical laboratory	Advantages: • Provides platelet function information using a small blood volume • Uses a variety of specific agonists which allows for a variety of applications • Yields results rapidly (within 15 min) • Allows for platelet function to be measured even at low platelet counts Disadvantages: • Is influenced by red cell count • Is manually conducted and may have significant operator variability • Provides unclear association of platelet function and bleeding risk
Clotting assays	aPTT, PT, TT (78–82)	• Clinical laboratory: 3 mL whole blood • Sample test volume: 1 mL of plasma	Clinical laboratory	Advantages: • Has common clinical use that is well-standardized and validated • Are helpful as a screening assessment for bleeding and thrombotic disorders • Are often utilized in diagnostic decision support and therapeutic algorithms • Are easy to perform either manually using the tilt-tube technique or automatedly using high throughput analyzers Disadvantages: • Are non-physiologic tests that oversimplify coagulation pathways • Are affected by *in vitro* and *in vivo* factors that do not have any effect on *in vivo* clot formation • Provides unclear assessment of bleeding risk
	Factor assays (78, 81)	• Clinical laboratory: 3 mL whole blood • Sample test volume: 0.5 mL of plasma	Clinical laboratory	Advantages: • Has common clinical use that is well-standardized and validated • Is useful in the diagnosis of specific protein alterations that may cause or contribute to bleeding and thrombosis disorders Disadvantages: • Requires a large blood volume for pediatric patients, particularly if testing multiple factor levels • Affected by *in vitro* and *in vivo* factors that do not have any effect on *in vivo* clot formation
Global measures	Viscoelastic testing (83–96)	• Clinical laboratory: 3 mL whole blood • Sample test volume: ≤ 400 μL whole blood	Point-of-care	Advantages: • Has rapid turn-around time and may be performed at the bedside • Is more cost-effective than standard measurements of hemostasis • May decrease use of blood products in certain populations Disadvantages: • Requires operator training in interpretation • Requires rapid processing of sample (within 3 min) for some measures • Has poor precision with high coefficient of variance • Is unclear in ability to predict bleeding risk
	Exogenous thrombin potential (97, 98)	• Sample test volume: ≤ 500 μL platelet-rich or platelet-poor plasma	Primarily research	Advantages: • Is reflective of the physiologic state, assessing activation, clot formation, and fibrinolysis • Allows for different antibiotics, proteins, and enzymes to interrogate different aspects of hemostasis Disadvantages: • Does not have fully established and validated reference ranges • Is not readily available for clinical use
	Sonic estimation of elasticity resonance (99–102)	• Sample test volume: 1.5 mL whole blood	Primarily research	Advantages: • Provides a direct estimate of mechanical clot properties Disadvantages: • Does not have fully established and validated reference ranges • Is not readily available for clinical use

*aPTT, activated partial thromboplastin time; PT, prothrombin time; TT, thrombin clotting time*.

### Platelet Evaluation

While the important role platelets play in the initiation and regulation of clot formation has been recognized for over 100 years, the ability to measure how well-platelets function in these processes remains limited (59). Initial evaluations were confined to determining platelet number and morphology in peripheral blood and did not provide any specific information regarding platelet function (62). However, with the development of light transmission aggregometry (LTA), direct assessment of how platelets respond and aggregate to agonists *ex vivo* was possible (63). While admittedly non-physiologic, this methodology has remained the cornerstone of platelet function assessment for the past 60 years (discussed further below).

Platelet enumeration can be performed in a variety of ways and remains a first-line screening test in platelet evaluation. In manual methods, platelets are observed under light microscopy and counted by hand, a process which is time consuming and carries a coefficient of variation between 15 and 25% (64). Automated counts are more typically utilized as they are rapid, reproduceable and require less tech time. However, while they are highly accurate in most circumstances, cellular debris may result in overestimation or underestimation of platelet number in samples with platelet clumping or in patients with enlarged platelets (6, 66). Optical counting methods, in which platelets are recognized by light scattering or fluorescent dye, increase accuracy by better measurement of different sized platelets and better discrimination of platelets from cellular debris and other cell populations (66). In flow cytometric methods, platelets are incubated with fluorescent monoclonal antibodies directed against an antigen on the platelet membrane. While determination of platelet number may be different depending on the analyzer, all have been shown to be accurate with platelet counts as low as 10,000/μL (64, 67, 68).

Despite these advances, all platelet counting methods may be problematic in severely thrombocytopenic patients, an inaccuracy that may have substantial clinical impact around bleeding assessment and transfusion. A common cause of artifactual underestimation of platelet count occurs when patient plasm contains EDTA reactive antibodies causing *in vitro* platelet clumping. Most clinical laboratories will identify the presence of platelet clumps or cellular debris and not report an actual platelet number due to the possibility of a spurious result. The clinician must be aware of this possibility when an unexpected high or low platelet count is reported.

Assessment of platelet function through measurement of *in vitro* platelet aggregation, developed in the 1960's, significantly improved the ability to identify alterations in platelet function (63). In this technique, referred to as Light Transmission Aggregation (LTA), platelet-rich plasma is stirred in a cuvette between a light source and a measuring photocell. Agonist-induced platelet aggregation results in increased transmission of light through plasma over time. Multiple agonists are commonly employed and trigger a highly characterized response for each agonist in normal platelets. Deviation from that agonist-specific response enables diagnosis of platelet disorders based on the profile of aggregation (69). In addition to aggregation, shape change, adenosine triphosphate secretion, and (with special equipment) an increase in cytoplasmic calcium ions can also be measured. While much information can be derived from this classical method, turbidimetric platelet aggregometry has several important barriers that limit its practical use. First, it is labor intensive and requires a significant degree of technical expertise to carry out (70, 71). Second, this method is restricted in its ability to assess small aggregates and therefore may have limited sensitivity to both preexisting aggregates or the early phase of aggregation (72). Third, platelet adhesion and change in surface expression of cytoadhesion receptors are also not assessed (70), and fourth, it requires large volume blood sample and (generally) a platelet count over 100,000–200,000/υL limiting its use in small infants and in those with severe thrombocytopenia.

Whole blood aggregometry is another type of LTA and variation of turbidimetric aggregometry. In this technique, whole blood is stirred between two platinum wire electrodes set at a fixed distance (73). The electrode becomes covered with platelets and further adhesion of platelet aggregates is induced after the addition of agonists all of which change the impedance with time (73). Whole blood aggregometry may be superior to turbidimetric aggregometry when monitoring anti-platelet therapy, but is similarly insensitive to smaller platelet aggregates (74). Light scattering methods analyze particle size by flow cytometry and increases the accuracy for counting platelets and for assessing aggregates of all sizes during the early aggregation phase (103). Light scattering may be combined with aggregometry to monitor the continued formation of platelet microaggregates (73). This combined technique accurately assesses primary and secondary aggregation responses and may be of specific use in platelet hyperreactivity disease states (104).

Bleeding time (BT) was the first, and remains one of the only, test to evaluate *in vivo* platelet function. Via the Duke, Ivy, or Mielke method, a “standardized” cut is created and the time until bleeding flow arrests is assessed (75, 76). For the pediatric population, there are commercially available devices based on the Mielke method with age-appropriate templates that take into account the thinner epidermis and size of newborns and infants. While still occasionally used as first line screening for severe hemostatic defects, the perioperative use of the BT has largely disappeared because it, along with many other platelet function tests, does not accurately predict the risk related to bleeding for surgical procedures unless grossly abnormal (77, 105). This suggests that either the test is insensitive or mild platelet defects are not clinically important. Advantages of bleeding time include study of platelet function *in situ* and the ability to perform the test without complex equipment. Disadvantages include its manual nature with variable reproducibility, the invasiveness of the procedure, and that it is time consuming (77, 105).

In addition to bleeding time, there are number of other tests that have been developed to mimic the processes that occur *in vivo* following vessel injury. The hemostatometer, a commercially available clot signature analyzer, punches holes within a tube containing flowing anticoagulated blood. Under controlled conditions, the punched holes stimulate formation of the primary platelet plug and allow for the assessment of platelet function and of involvement of GPIb, GPIIb/IIIa, and vWf (19, 106). The thrombotic status analyzer pulls whole blood through a capillary tube and the resulting force induces platelet activation, formation of the platelet plug, and eventually capillary tube occlusion (107). The platelet function analyzer (PFA-100 or updated PFA-200) exposes citrated whole blood to high shear in a capillary tube with a center membrane coated with an agonist. As platelets are activated and aggregate, the device measures the drop in flow rate between the capillary tube and center membrane and ultimately measures tube occlusion or closing time (78, 105). While referred to as an *in vitro* bleeding time, its ability to identify platelet disorders is poor and currently this test may serve as a test for screening and monitoring of von Willebrand disease in the pediatric population (78, 79). The cone and plate analyzer exposes a whole blood sample to a plate under arterial flow conditions and assesses platelet adhesion and aggregation on the surface of the plate (80, 105). For the pediatric population, the main advantage of this system is that it requires only a small volume of blood to perform (150–250 μL) (80). Platelet mapping can be performed using thromboelastography (TEG) though its clinical utility and correlation with other platelet function assays has not been determined (81). In general, none of these tests have been shown to have clinical utility in the routine assessment of platelet function.

Finally, platelet function may be assessed through the identification of markers of platelet activation and reactivity in plasma and/or on circulating platelets. This may be evaluated through measurement of platelet release products such as platelet factor 4 and β-thromboglobulin in platelet-poor plasma. Whole blood flow cytometry can be used to measure activated platelets, platelet hypo-reactivity or hyper-reactivity, platelet-leukocyte aggregates, platelet microparticles, and platelet turnover which is inferred through the measurement of reticulated (i.e., young) platelets (82). Nucleic acid-specific dyes can be used to measure young platelets that contain residual RNA and aid in the assessment of platelet turnover or thrombopoiesis (83).

### Clotting Assays

The most commonly utilized tests measuring the effectiveness of *in vivo* clotting measure the conversion of fibrinogen to fibrin and utilize the time to formation of a fibrin clot as determined by one of several methodologies each of which have a different degree of sensitivity. The most common of these tests are PT, aPTT, and thrombin time (TT) (84). A few important aspects of nomenclature for these tests should be noted. First, in contrast to the older PTT, aPTT is an updated, modified, test that includes the addition of an activator to accelerate clotting time resulting in a narrower reference range and increased sensitivity (84, 85). Second, because it is calculated from PT, International Normalized Ratio (INR) is frequently employed as a surrogate PT. However, INR has only been validated as a measure of intensity of vitamin K antagonist induced anti-coagulation and has not been validated as a measure of bleeding risk (86). While the Activated Clotting Time (ACT) is generally performed to monitor anticoagulation during cardiopulmonary bypass surgery and during ExtraCorporeal Membrane Oxygenation (ECMO), outside of these clinical settings it has not gained widespread use.

PT, aPTT, and TT are reported as time to clot formation while fibrinogen is reported as mg/dL determined by a lab-specific calibration curve (84, 87). In regards to assays utilized for the quantification of protein levels of specific clotting assays, time to clot formation is measured and then reported as a percent in relation to time to clot formation using pooled normal adult plasma. By convention, the amount of a specific clotting factor contained in normal plasma is defined as 100%, equivalent to 1 unit/mL in normal plasma. From this assumption, time to clot formation is converted to a U/mL concentration. All of these assays where the end point is the time to a fibrin clot are referred to as “functional assays.” An advantage of these assays is that they are relatively simple and easy to standardize, but they can be affected by *in vitro* and *in vivo* factors that do not have any effect on *in vivo* clot formation (84, 87). One such circumstance is common in neonates, particularly premature neonates, and in infants with cyanotic heart disease who manifest a reactive polycythemia with hematocrit >55%. Under this condition, blood collected into standard coagulation testing vacuum tubes (for example, “blue top tubes”) may produce PT or aPTT with values that are spuriously prolonged as these samples contain less plasma and are therefore over-anticoagulated (88). This can be remedied by collecting blood in tubes that contain a reduced amount of anticoagulant.

### Global Measures of Hemostasis

There are several important global measures of hemostasis that are gaining use and clinical application. This includes viscoelastic testing, a method of assessing clot formation dynamically in whole blood. Viscoelastic testing is designed to imitate sluggish venous blood flow and triggers clot formation to derive measurements of hemostasis (89). Developed in 1948, but not adopted in the clinical setting until the 1980's, viscoelastic testing now has more broad use primarily in trauma, surgery, and cardiopulmonary bypass (89–91).

Currently available methods for viscoelastic testing include thromboelastography (TEG) and the related rapid thromboelastography (r-TEG) as well as rotational thromboelastography (ROTEM) (89). In both, a small sample of patient blood is placed into a cup and a pin that serves as a sensor is inserted. In TEG and r-TEG, the cup is oscillated with a specified rotational cycle to precipitate clot formation. With clot formation, the torque of the rotating cup is transmitted to the pin and the degree of pin rotation is converted to an electrical signal and recorded. The device tracing reflects this change in electrical signal reflecting change in clot strength resulting from clot formation or fibrinolysis. ROTEM uses similar methods but the sensor pin instead rotates within the cup (91).

Viscoelastic testing measures clot initiation or time to initial clot formation, kinetics or growth of the clot, clot strength, and clot lysis. All components are identified by specific measurements on a tracing produced by the device over time. Clot initiation or clot time provides an assessment of early activity of the clotting cascade before clot amplification and the burst of thrombin production. Clot kinetics or clot formation time assesses clot potentiation by activation of platelets and thrombin-mediated cleavage of soluble fibrinogen. Clot strength describes maximal clot strength achieved via GPIIb/IIIa-mediated platelet-fibrin interactions. Finally, clot lysis provides an assessment of activation of the fibrinolytic system (89, 92, 93). In addition to standard viscoelastic testing, specific elements of the clotting cascade can be interrogated by the addition of specific clotting activators or inhibitors (94).

The diagnostic and prognostic role of viscoelastic testing is being studied in a range of clinical areas (89, 92, 93). The majority of these studies have been in adult perioperative cardiac surgery patients as well as other surgical patients at risk for hemorrhage (89). A recent Cochrane review of 17 studies in a largely cardiac surgery population, only 2 of which included pediatric patients, addressed the utility of thromboelastic testing in bleeding patients (95). They found that while TEG/ROTEM-guided transfusion strategies might decrease transfusion requirements and support a tendency toward improved mortality outcomes, conclusive recommendations could not be made because of limitations in study design and power (88). Subsequent studies in pediactric patients (largely neonates) has continued to investigate the utility of viscoelastic testing as a modality to better manage patients with acquired coagulopathies or requiring anticoagulation (96, 97). Reference range values have been established in term, preterm and very low birth weight infants (98, 99). In the trauma population, TEG-guided resuscitation is growing in use, though current studies are insufficient to determine if thromboelastography-based transfusion is better than existing transfusion practices (100, 101). Other populations of potential interest include liver transplantation, sepsis, obstetric hemorrhage, perioperative thromboembolism, and management of anticoagulation on mechanical circulator support, though evidence from large trials required to formulate consensus recommendations in these areas is lacking (89). This work is even more limited in pediatric specific populations, though studies have suggested a benefit in similar disease populations as to adults (102, 108). That said, viscoelastic testing may be particularly advantageous in pediatric populations since only a small volume of blood is required for sample analysis. Further, in limited use TEG has been shown to be reproducible in some infant populations, and cost-effectiveness studies seem to clearly favor viscoelastic testing compared to standard laboratory run measures of hemostasis (102, 109–113). Taken together, viscoelastic testing remains a novel method that may be advantageous in certain populations including children.

Endogenous thrombin generation is a measure of the global efficiency of coagulation as thrombin sits at the key intersection of endothelial cell, platelet, and coagulation activation, clot formation, and fibrinolysis. Thrombin generated can be quantified in platelet-rich or platelet-poor plasma using the calibrated automated thrombogram (CAT) method. Specifically, CAT monitors the cleavage of a fluorogenic substrate that is compared to the known thrombin activity in a non-clotting plasma sample (100). The CAT system is open, allowing for different antibiotics, proteins, and enzymes to be introduced to evaluate specific aspects of hemostatic process (114). Validation of reference ranges are just now being established with preliminary studies suggesting children and adolescents have lower exogenous thrombin potential compared to adults (115); although as previously stated, low thrombin generation potential noted in infants may reflect in part how the assay is performed (58). While these assays have great clinical potential, standardization and validation need refining as does a more rapid technical process.

Another potentially promising method is sonic estimation of elasticity via resonance. This technology applies acoustic radiation force to induce shear wave resonance within a rigid test chamber. The characteristics of this resonance are compared to numerical or analytical models to quantify the sample filling the chamber (116, 117). The technique may be performed rapidly and shows promise as a point-of-care test and may rival TEG/ROTEM in assessment of clot stiffness and fibrinogen contribution (117–119).

## Special Considerations

There are a number of additional considerations in the assessment of hemostasis in the pediatric population. First, there are numerous technical challenges. This includes practical difficulty drawing sufficient blood volume from small infants and children given their small vessels for certain tests (e.g., LTA) and the overall smaller total blood volume infants and children which limits the quantity of blood that can safely be obtained to perform comprehensive diagnostic testing. Consequently, tests that use small volumes may be preferred and careful consideration is necessary to parsimoniously select appropriate tests given the clinical situation. While central lines help with the technical difficulties of obtaining adequate testing volumes, samples can be contaminated by anticoagulation or other medications running through the line itself. In fact, samples collected through central lines may require 10 mL of blood be withdrawn prior to sample collection for the result to be considered valid. Techniques to mitigate this waste include using the initial sample collection for other labs or reinfusing blood that would otherwise be discarded.

There are also a number of infant and pediatric conditions and diseases that require special testing considerations. Pediatric trauma patients, particularly infants, demonstrate significant endothelial injury, present with acute traumatic coagulopathy and/or coagulopathy related to iatrogenic causes, and, while they may be at less risk of bleeding than their adult counterparts, they are also at risk of thrombosis during recovery from their injury (120–123). While there have been several pediatric-focused studies around damage control resuscitation and thromboelastographic-guided transfusion, there are still gaps in how these tests account for the developmental changes in hemostasis noted in infants and children (99, 116). Pediatric patients with liver dysfunction may have significant hemostatic abnormalities from impaired synthetic function including coagulation factor defects, thrombocytopenia and platelet dysfunction, alterations in the fibrinolytic system, and alterations in endogenous inhibitors of coagulation. While clotting times may be significantly elevated in these patients, these abnormal results often do not correlate with bleeding risk (124, 125). Indeed, assessment of hemostatic balance in patients with liver disease generally demonstrates a neutral or mildly pro-thrombotic hemostatic system (126, 127). The concept of “hemostatic balance” has also been conceptually addressed as alternatively being a condition of a loss in “hemostatic reserve” by some hematologists, a concept that carries some merit (128). In the oncologic population, patients may be at risk of hyperviscosity, thrombosis, and cytopenias that may increase bleeding risk depending on the type of cancer and therapies. The hemostatic system is frequently monitored in these patients, but transfusion and other therapeutic decisions necessitate an understanding of the limitations and normal variations of these standard hematologic measurements (129).

Patients on extracorporeal life support present with unique hemostatic risks as both the extracorporeal circuit and the underlying disease process induce important dysregulation of the hemostatic system that may favor either hemorrhage or thrombosis (119, 120). Hematologic monitoring of these patients may be one of the most active parts of their management and typically includes frequent evaluation for bleeding, consumptive coagulopathy, hemolysis, and adequacy of anticoagulation. In addition to aPTT to assess anticoagulant effect of unfractionated heparin (UFH), anti-Xa assay (assessing effect of UFH or low molecular weight heparin [LMWH] or some of the new direct acting oral anticoagulants [DOACs]) or the Activated Clotting Time (ACT) are utilized to assess bleeding/thrombosis risks (130, 131) Increasingly, the anti-Xa assay is becoming the preferred test to monitor UFH as well as anti-Xa inhibitors due to its insensitivity to low levels of AT. However, therapeutic range for anti-Xa has been extrapolated from those for UFH by matching aPTT result to anti-Xa level in plasma samples to which heparin was added *ex vivo*. This practice does not account for the primarily anti-IIa action of UFH potentially introducing a degree of uncertainty regarding the extrapolated therapeutic threshold (132, 133). ACT is the preferred monitoring test for UFH given during cardiopulmonary bypass surgery with “therapeutic” (i.e., desired) values being surgeon and device specific (134, 135).

Finally, trauma patients often present in the field or to community hospitals and must be transported to the regional trauma center for care. The use of point of care (POC) tests of hemostasis have only recently been investigated in this patient population (136, 137). While some studies suggest the feasibility of these devices, there are as of yet insufficient data to adequately assess their use.

## Conclusions

Understanding of hemostatic dysfunction remains a unique challenge in critically ill infants and children. It requires knowledge of physiologic hemostatic pathways, comprehension of the development of the hemostatic system by age, an understanding of logistics, advantages, and limitations in hematologic testing, and an awareness of the specifics of application to pediatric populations. Taken together, this knowledge will enable the practitioner to more accurately assess hemostatic dysfunction and determine bleeding and thrombosis risk resulting in more successful diagnosis and management of this vulnerable population.

## Author Contributions

AN drafted all tables included. RP created all figures included in manuscript. Both authors participated in reviewing appropriate published literature, writing all drafts, and the final submitted draft manuscript.

## Conflict of Interest

The authors declare that the research was conducted in the absence of any commercial or financial relationships that could be construed as a potential conflict of interest.
